# Circular RNA circNCOA3 promotes tumor progression and anti-PD-1 resistance in colorectal cancer

**DOI:** 10.20517/cdr.2023.151

**Published:** 2024-03-13

**Authors:** Dong-Liang Chen, Nuo Chen, Hui Sheng, Dong-Sheng Zhang

**Affiliations:** State Key Laboratory of Oncology in South China, Guangdong Provincial Clinical Research Center for Cancer, Sun Yat-sen University Cancer Center, Guangzhou 510060, Guangdong, China.

**Keywords:** circRNA, circNCOA3, colorectal cancer, anti-PD-1 therapy, immune evasion

## Abstract

**Aim:** Circular RNAs (circRNAs) have been found to be involved in tumor progression, but their role in colorectal cancer (CRC) immune escape remains to be elucidated.

**Methods:** circRNAs differentially expressed in responsive and resistant CRC tissues to programmed cell death 1 (PD-1) antibody therapy were identified by microarray analysis. The clinical and pathological significance of circNCOA3 was validated in a separate cohort of CRC samples. The function of circNCOA3 was explored experimentally. RNA immunoprecipitation and luciferase activity assays were conducted to identify downstream targets of circNCOA3.

**Results:** The circNCOA3 was markedly overexpressed in CRC samples resistant to PD-1 blockade. circNCOA3 expression was significantly correlated with adverse tumor phenotypes and poor outcomes in CRC patients. Knockdown of circNCOA3 expression markedly suppressed the proliferative and invasive capability of CRC cells. Moreover, knockdown of circNCOA3 increased the proportion of CD8^+^ T cells while decreasing the proportion of myeloid-derived suppressor cells (MDSCs). Knockdown of circNCOA3 inhibited tumor growth and increased the sensitivity to PD-1 antibody treatment in mouse tumor models. Further studies revealed that circNCOA3 acted as a competing endogenous RNA (ceRNA) for miR-203a-3p.1 to influence the level of CXCL1.

**Conclusion:** Our findings indicate that circNCOA3 might be useful as a potential biomarker to predict the efficacy and prognosis of CRC patients treated with anti-PD-1 therapy.

## INTRODUCTION

Colorectal cancer (CRC) is one of the most commonly diagnosed tumors and a leading cause of cancer-related deaths in the world^[1]^. Thanks to the development of novel treatment strategies in recent years, the mortality rate of CRC is declining. However, the prognosis of CRC is still unsatisfactory, especially in patients with advanced stages or old age^[2]^. Currently, the main treatment option for advanced CRC patients is chemotherapy combined with targeted therapy^[3]^. However, drug resistance is common in CRC and affects the therapeutic efficacy^[4,5]^. A growing body of literature indicates that escape from immune surveillance is critical for cancer growth and progression^[6]^. Clinically, it has been confirmed that immune checkpoint inhibitors, especially programmed cell death 1 (PD-1) antibody therapy, are effective in various cancers^[7,8]^. For CRC, anti-PD-1 therapy is efficacious in metastatic microsatellite-instability-high (MSI-H) or mismatch-repair-deficient (dMMR) tumors^[9-11]^. The KEYNOTE-177 phase III clinical trial proved the effectiveness of PD-1 antibody therapy in first-line treatment of CRC. Nevertheless, about 30% of MSI-H/dMMR CRC patients display primary resistance to PD-1 antibody treatment^[11]^. Among patients with microsatellite stable (MSS) CRC, the objective response rate (ORR) to PD-1 antibody treatment is nearly 0%^[12]^, and it could be increased with the combination of Regorafenib. Previous studies explored the association of some factors with primary resistance, including a relatively low tumor mutational burden, elevated systemic inflammation, heterogeneity of MSI-H/dMMR, and tumor intrinsic metabolic reprogramming^[13-16]^. However, the underlying molecular mechanism of anti-PD-1 resistance in CRC remains virtually unknown.

Circular RNAs (circRNAs) were identified as a novel type of non-coding RNAs or protein-coding RNAs with covalently closed loops and lack 5’ to 3’ polarity or polyadenylation tail^[17]^. circRNAs generally arise from alternative back-splicing of pre-mRNA transcripts and are stable, conserved, and expressed in multiple cancer cells and tissues^[18]^. circRNAs have been found to be involved in various aspects of tumor formation, such as proliferation and metastasis^[19,20]^. Recent studies found that circRNAs participate in tumor immune microenvironment regulation and mediate PD-1 antibody responsiveness^[21]^. However, the role of circRNAs in CRC immune regulation and PD-1 antibody responsiveness has not been established.

## METHOD

### Cell lines and tissues

For RNA microarray and real-time PCR analysis, paraffin-embedded or fresh-frozen tissues, or serum were obtained from metastatic CRC patients who underwent colonoscopy with biopsies or surgery and PD-1 inhibitor treatment at Sun Yat-sen University Cancer Center (SYSUCC) between July 2018 and May 2022. Response rates were determined based on the RECIST 1.1 guideline. The study was approved by the ethics committee of the SYSUCC, and informed consent was obtained from each patient. Progression-free survival (PFS) and overall survival (OS) were assessed from the date of PD-1 antibody therapy to the date of progression (PFS) or death from any cause or last contact (OS).

CRC cell lines (HT29, SW620, HCT116, SW480, DLD-1, LoVo), normal colon epithelial cell NCM460, and mouse MC38 colon cancer cells and HEK 293T cells were obtained from the cell bank of the Shanghai Institute of Cell Biology (Shanghai, China). The cell lines were maintained and cultured following the supplier’s protocols.

### circRNA microarray and quantitative real-time polymerase chain reaction, RNA immunoprecipitation, fluorescence *in situ* hybridization assays, and luciferase activity

The circRNA microarray, real-time polymerase chain reaction (RT-qPCR), fluorescence *in situ* hybridization (FISH), RNA immunoprecipitation (RIP), and luciferase activity experiments were conducted according to methods described previously^[22]^ and in Supplementary Methods. The primers are presented in Supplementary Table 1.

### Immunohistochemistry analysis and Western blot

The immunohistochemistry (IHC) procedure was conducted following our previously described protocols^[23]^. The Western blot (WB) was conducted as previously described^[24]^.

### Cell proliferative and cell invasive capability assay

Cell proliferation was determined using a cell counting kit-8 (CCK-8) and colony formation experiments. Transwell experiment was performed to determine cell invasive ability. These experiments were performed following a procedure described previously^[22]^ and in the Supplementary Methods.

### Flow cytometry analysis

Flow cytometry (FC) analysis was performed following the procedure described before^[25]^ and in the Supplementary Methods. Information on antibodies is presented in Supplementary Table 2.

### Knockdown of circNCOA3 transfection

circNCOA3 stable knockdown lentivirus was obtained from Geneseed Biotech Co., Ltd (Guangzhou, China). For lentiviral production, HEK293T cells were co-transfected with the lentiviral vectors (or the control lentiviral vectors) with Lenti-Pac HIV Expression Packaging Mix using Lipofectamine 2000 (Life Technologies Corporation, Carlsbad, CA, USA). After 48 h, supernatants containing the lentiviral particles were collected and centrifuged at 500 *g* for 10 min, followed by filtration using a 0.45 µm filter. MC-38 cells were then transfected either with sh-circNCOA3 lentivirus or with the control virus (sh-NC). The virus-transduced cells were identified by GFP fluorescence. Stably transfected cells were selected using puromycin (2 μg/mL) for 2 weeks and then expanded for subsequent assays.

### Tumorigenesis assay

To evaluate the role of circNCOA3 on *in vivo* tumor growth, the *in vivo* tumorigenesis assay was carried out following the method described previously^[25]^ and in the Supplementary Materials. Experimentation on mice was approved by the Committee on the Ethics of Animal Experiments of SYSUCC.

### Mice xenograft PD-1 antibody treatment

Xenograft experiments were performed using C57BL/6 mice to assess the role of circNCOA3 in influencing the efficacy of PD-1 antibody treatment. Briefly, a total of 1 × 10^6^ cells (MC38-sh-circNCOA3 or MC38-sh-NC) were implanted subcutaneously in the left flank of 6-week-old C57BL/6 mice. Each mouse population was randomly divided into two groups and injected intraperitoneally with either PBS or PD-1 antibody (Bio X Cell, West Lebanon, NH, USA) according to a previously described method^[26]^.

### Statistical analysis

All the data are presented as mean ±standard deviation unless otherwise noted. The statistical analysis was conducted by using GraphPad Prism 7 or SPSS 17.0 software. Student’s *t*-test, one-way ANOVA, chi-squared test, and Pearson correlation analysis were performed where appropriate. The Kaplan-Meier method was used to assess OS and PFS using the log-rank test. A *P* value of < 0.05 was considered statistically significant.

## RESULTS

### circRNAs that are correlated with responsiveness to PD-1 antibody therapy of CRC

**Figure 1:**
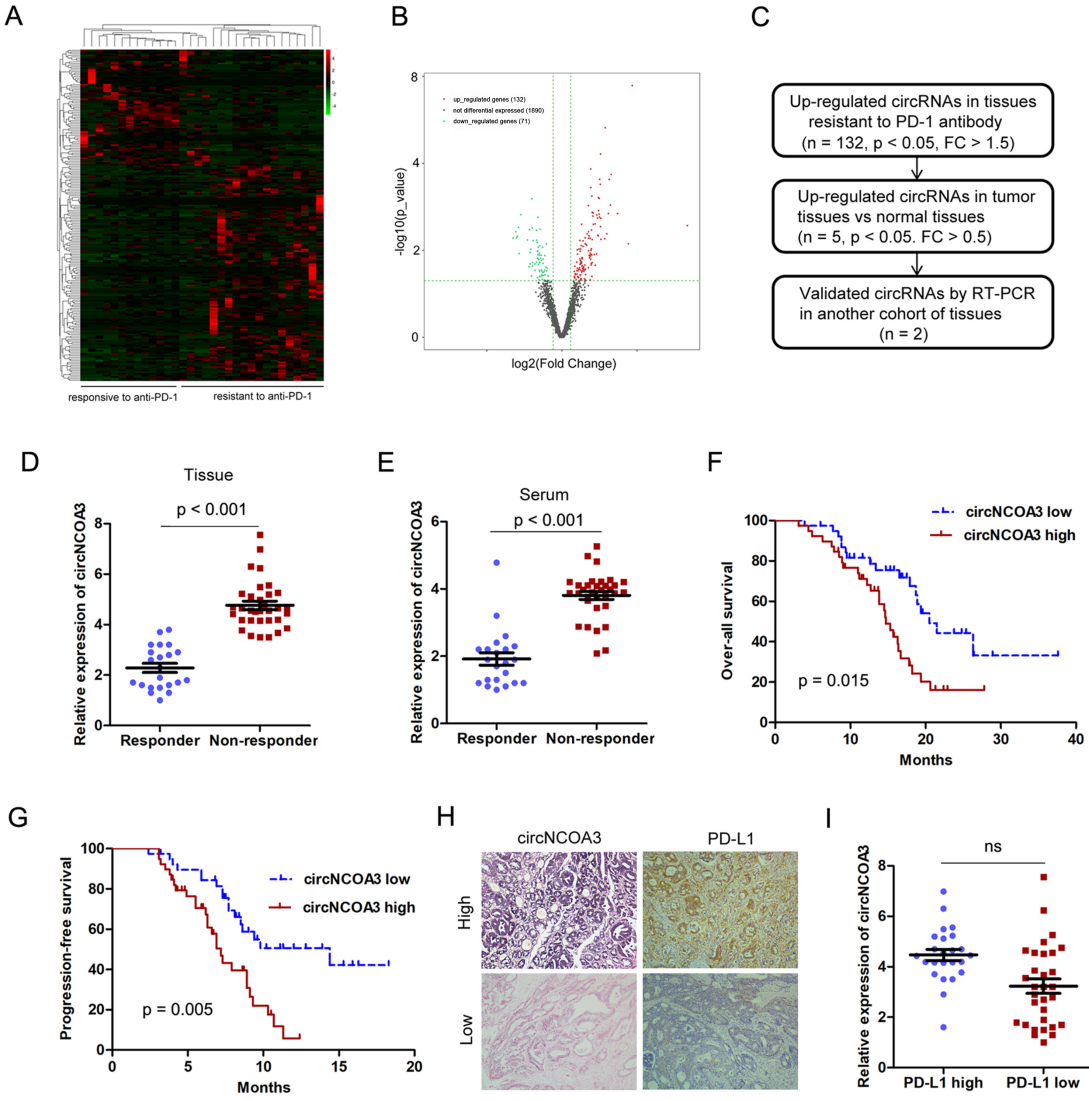
Identification of circRNAs associated with responsiveness or resistance to PD-1 antibody therapy of CRC. (A) Heat map showing the differentially expressed circRNAs in CRC samples responsive (*n* = 13) or resistant (*n* = 19) to PD-1 antibody treatment analyzed by circRNA microarray; (B) Volcano plot showing circRNAs differentially expressed in tissues responsive or resistant to PD-1 antibody treatment. Cut off is Log 2 (Fold Change) > 1.5, *P* < 0.05; (C) Procedure to select the key circRNA involved in CRC progression and tumor immune evasion; (D and E) Relative expression of circNCOA3 in primary CRC tissues (D) and in the serum (E) of patients responsive (*n* = 22) and resistant (*n* = 33) to PD-1 antibody treatment in a separate cohort (*P* < 0.001); (F and G) Kaplan-Meier analysis of the association of circNCOA3 expression level with OS (F) and PFS (G) in CRC patients treated with PD-1 antibody; (H) Representative pictures of circNCOA3 expression (detected by ISH) and PD-L1 expression (detected by IHC) in CRC tissues; (I) Expression of circNCOA3 in the PD-L1 high and PD-L1 low expression groups. circRNAs: Circular RNAs; PD-1: programmed cell death 1; CRC: colorectal cancer; OS: overall survival; PFS: progression-free survival; IHC: immunohistochemistry.

During the period from July 2018 to May 2022, 32 patients were diagnosed with CRC (20 with MSS and 12 with MSI-H) and were provided with PD-1 antibody-based treatment. All patients received at least two cycles of PD-1 antibody treatment and were evaluated for efficacy by computed tomography (CT) and tumor-related markers. Among these patients, 2 patients obtained complete response (CR), 11 patients obtained partial response (PR), 12 patients remained stable disease (SD), and 7 patients exhibited progressive disease (PD). The ORR was 40.6% and the disease control rate (DCR) was 78.1%. To identify the circRNAs that might be associated with CRC immune evasion and anti-PD-1 efficacy, we conducted circRNA microarray analysis using 32 CRC samples (13 responsive and 19 resistant to PD-1 antibody treatment). A total of 2,118 circRNAs were detected, the majority with a length of 50-500 nt. Most of the reads mapped to exons. The circRNAs differentially expressed in CRC samples resistant or responsive to anti-PD-1 therapy are presented in the heat map [Figure 1A]. Among these, 203 circRNAs were differentially expressed, out of which 132 were up-regulated and 71 were down-regulated in tissue resistant to PD-1 antibody therapy [Figure 1B]. In order to identify the circRNA that is critical for CRC immune evasion and anti-PD-1 efficacy and tumor progression, the circRNAs up-regulated in both anti-PD-1 resistant and tumor tissues were validated by RT-PCR analysis [Figure 1C]. Only has_circ_0060627 (circNCOA3) was confirmed to be up-regulated in tissues and serum of patients resistant to PD-1 antibody treatment in a separate cohort [Figure 1D and E]. A high circNCOA3 level was correlated with tumor size and liver metastasis, but was not associated with age, gender, tumor cell differentiation, and tumor location [Supplementary Table 3]. In addition, a high circNCOA3 level was correlated with poor OS and PFS of CRC patients who received PD-1 antibody treatment [Figure 1F and G]. However, there was no correlation between the expression of circNCOA3 and PD-L1 [Figure 1H and I].

### Characterization of circNCOA3 in CRC

**Figure 2:**
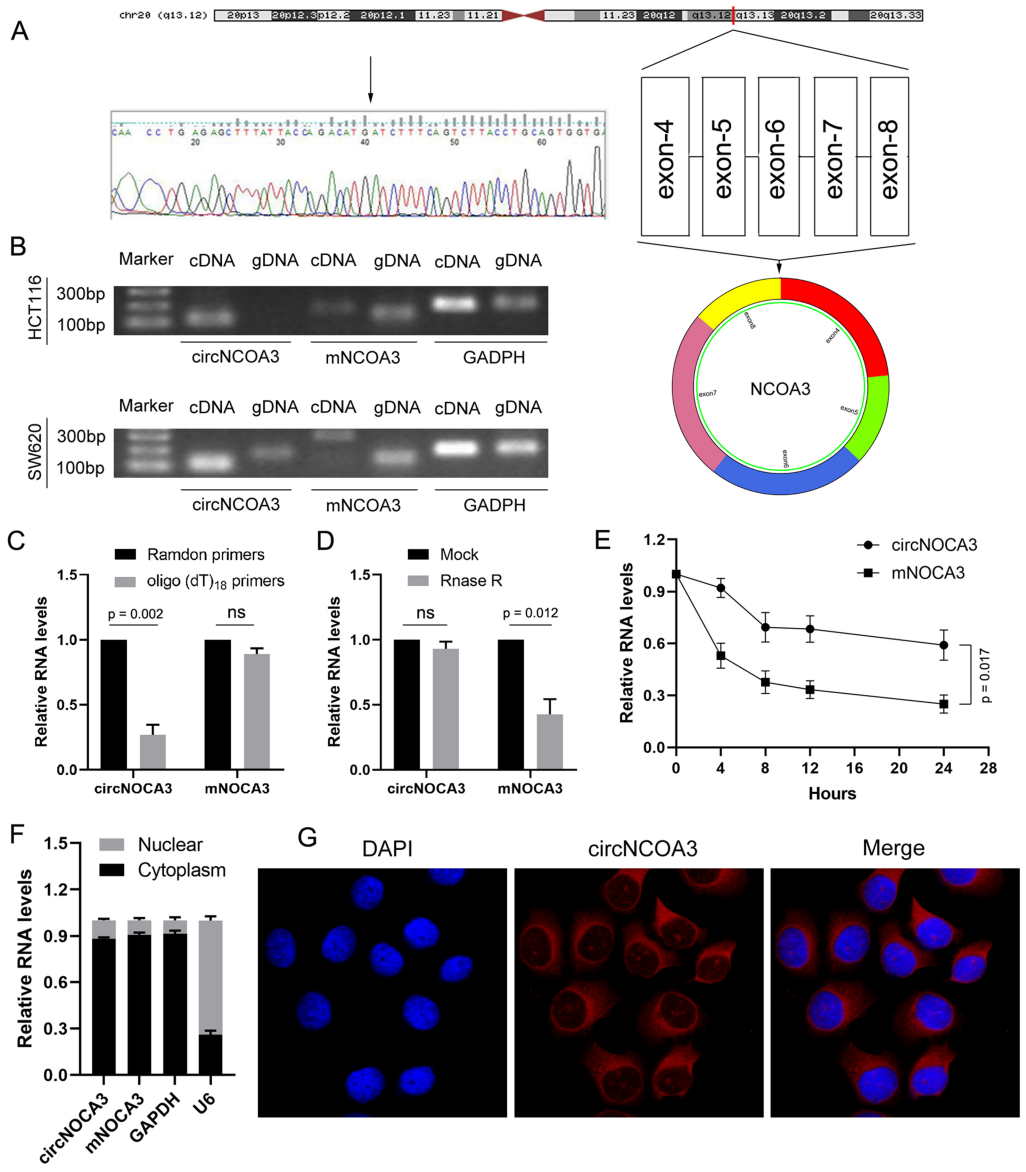
Characterization of circNCOA3 in CRC. (A) The genomic structure and back-splice junction site of circNCOA3 were analyzed using circBase database and Sanger sequencing; (B) The presence of circNCOA3 was confirmed in CRC cell lines by RT-PCR; (C) The relative RNA levels were analyzed by RT-qPCR using random hexamer or oligo (dT)18 primers; (D) Resistance of circNCOA3 and mNCOA3 to digestion with RNase R exonuclease was detected by RT-qPCR; (E) Abundances of circNCOA3 and mNCOA3 analyzed by RT-qPCR after treatment with actinomycin D at the indicated time points in CRC cells; (F) Cytoplasmic and nuclear RNA purification confirmed that circNCOA3 and mNCOA3 are abundant in the cytoplasm of CRC cells; (G) RNA FISH for circNCOA3. Nuclei were stained with DAPI. CRC: Colorectal cancer; RT-qPCR: real-time polymerase chain reaction; FISH: fluorescence *in situ* hybridization.

According to the UCSC and circBase database, has_circ_0060627 arises from exons 4, 5, 6, 7, and 8 of NCOA3 (circRNA ID: has_circ_0060627, chr20: 46252654-46256767), back-spliced with exon 4 and exon 8 (740 bp) [Figure 2A], and thus we designated it as circNCOA3. circNCOA3 was amplified by PCR using divergent primers and confirmed using Sanger sequencing [Figure 2A]. PCR analysis was performed using genomic DNA (gDNA) and reverse-transcribed RNA (cDNA), and the results indicated that divergent primers amplified circRNA in cDNA but not in gDNA [Figure 2B]. For validation of the circular status of the circNCOA3, RNA was reversely transcribed from CRC cells using random hexamers or oligo (dT)_18_ primers. The relative expression of circNCOA3, but not NCOA3 mRNA (mNCOA3), was markedly decreased when the primers were replaced by oligo (dT)_18_ [Figure 2C], indicating that circNCOA3 has no poly-A tail. circNCOA3 was resistant to digestion by RNAase R, a highly processive 3’ to 5’ exoribonuclease catalyzing the degradation of linear RNAs but not circular RNAs, whereas NCOA3 mRNA was not [Figure 2D]. Moreover, when cells were cultured with actinomycin D to inhibit transcription, the expression of circNCOA3 and mNCOA3 was detected at different time points by RT-qPCR, demonstrating that circNCOA3 has a significantly longer half-life than mNCOA3 [Figure 2E]. Besides, RT-qPCR and FISH results revealed that circNCOA3 is predominantly localized in the cytoplasm [Figure 2F and G]. Taken together, the data indicated that circNCOA3 is a stable and abundant circular RNA in cells.

### CircNCOA3 stimulates CRC development via regulation of the tumor immune environment

**Figure 3:**
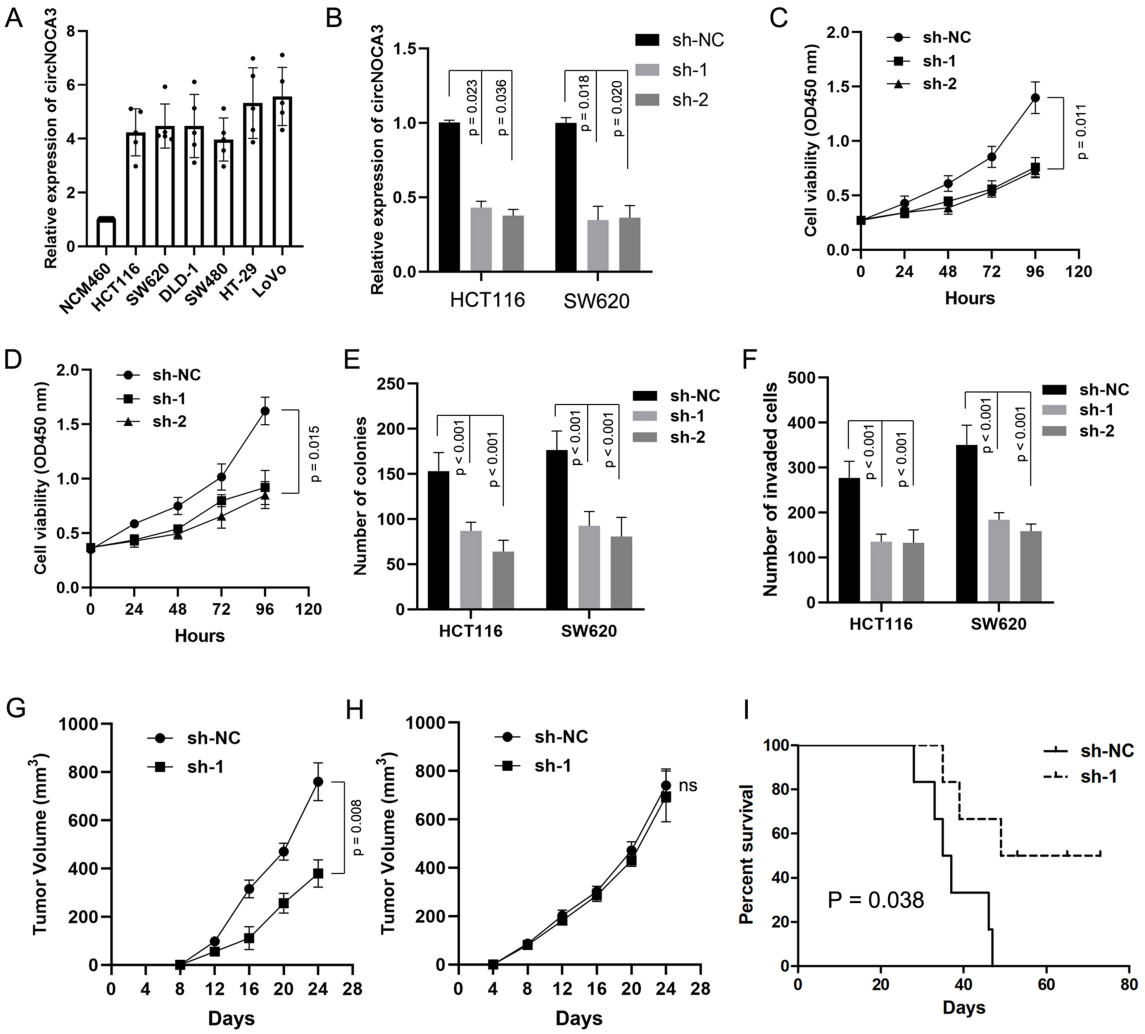
circNCOA3 promotes CRC progression. (A) Levels of circNCOA3 were measured by RT-qPCR in normal colon cells (NCM460) and various CRC cell lines; (B) Levels of circNCOA3 in CSC cell lines HCT116 and SW620 transfected with shRNAs; (C and D) Proliferation assays in cell lines HCT116 (C) and SW620 (D) transfected with sh-circNCOA3; (E) Colony formation assays of the CRC cell lines HCT116 and SW620 transfected with sh-circNCOA3; (F) Transwell assay of the CRC cell lines HCT116 and SW620 transfected with sh-circNCOA3; (G) Tumor growth in C57BL/6 mice implanted with MC38-sh-NC or MC38-sh-circNCOA3 cells; (H) Tumor growth in BALB/c nude mice implanted with MC38-sh-NC or MC38-sh-circNCOA3 cells; (I) The OS in different groups analyzed using the Kaplan-Meier method. CRC: Colorectal cancer; RT-qPCR: real-time polymerase chain reaction; OS: overall survival.

**Figure 4:**
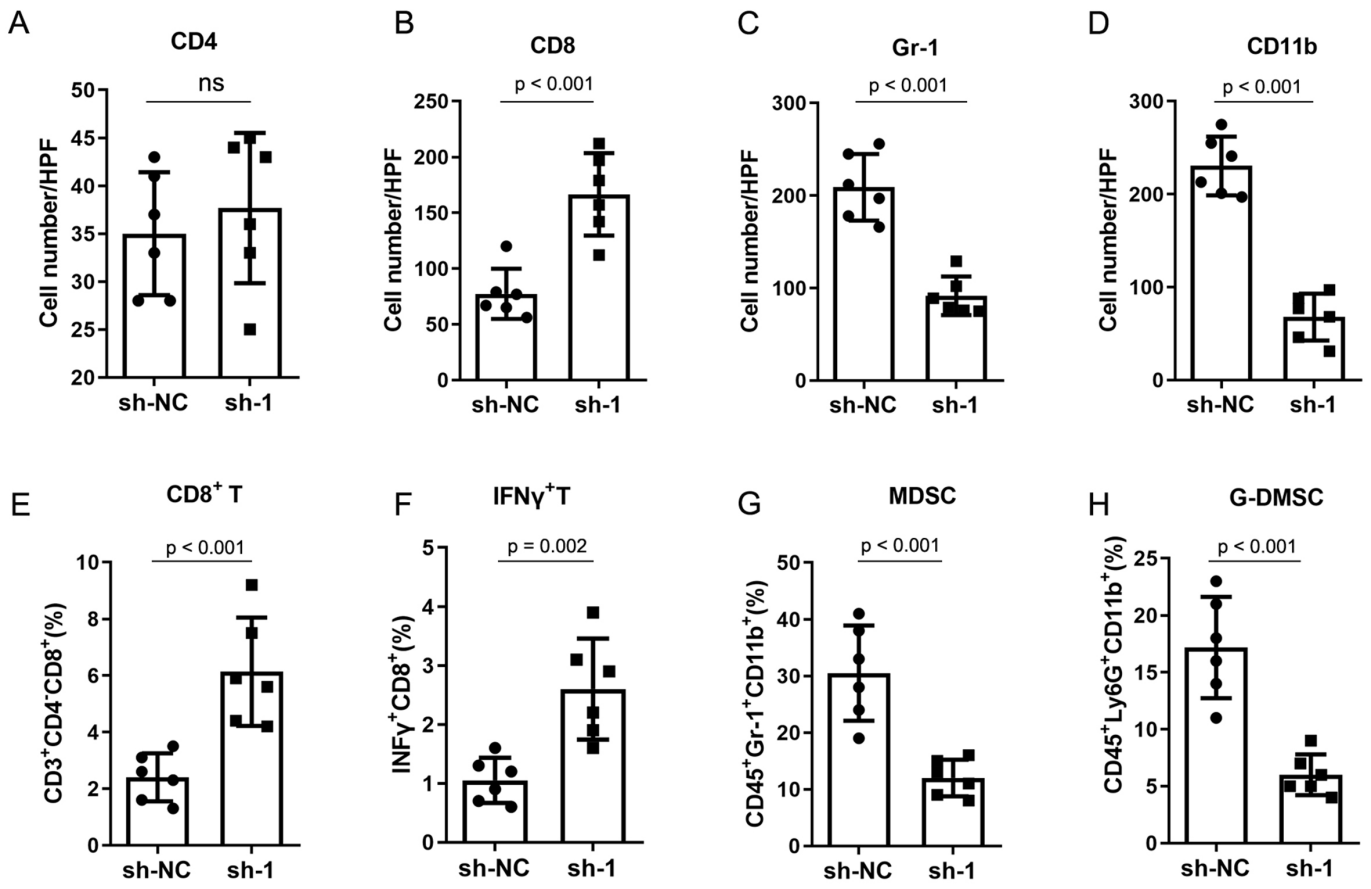
CircNCOA3 regulates the tumor environment of CRC in mouse models. (A-D) IHC analysis for CD4^+^ T cells, CD8^+^ T cells, and MDSC (Gr-1^+^CD11b^+^) from subcutaneous tumors; (E-H) FC analysis of immune cells from subcutaneous tumors in different groups. sh-NC: Negative control; sh-1, sh-circNCOA3; CRC: colorectal cancer; IHC: immunohistochemistry; MDSC: myeloid-derived suppressor cell; FC: flow cytometry.

To evaluate the biological role of circNCOA3 in CRC, the level of circNCOA3 was determined in CRC cells and normal colon cells NCM460. The results showed that circNCOA3 was up-regulated in all tumor cell lines compared to normal cells [Figure 3A]. When HCT116 and SW620 cells were transfected with specific shRNA plasmids targeting the unique back-splice junction of circNCOA3, the expression of circNCOA3 was significantly knocked down [Figure 3B]. The CCK-8 assay showed that the cell viability was markedly reduced in CRC cells after the knockdown of circNCOA3 [Figure 3C and D]. Colony formation assay revealed that cell proliferation was significantly suppressed in CRC cells after circNCOA3 knockdown as indicated by reduced colony formation numbers [Figure 3E]. In Transwell experiments, the cell invasive ability of CRC cells was significantly suppressed after circNCOA3 knockdown [Figure 3F]. To investigate the *in vivo* role of circNCOA3, we first generated MC38-sh-circNCOA3 cell lines by transfecting circNCOA3-targeting shRNA vectors into the MC38 cell line. MC38-sh-circNCOA3 cells and control cells were injected subcutaneously into immunocompetent mice (C57BL/6 mice) or immunodeficient mice (BALB/c nude mice). The results showed that tumor growth was significantly inhibited in immunocompetent mice after knockdown of circNOCA3 [Figure 3G]. However, no obvious difference in tumor growth was observed in immunodeficient mice after circNOCA3 knockdown [Figure 3H]. Moreover, the survival time was significantly longer after circNOCA3 knockdown in immunocompetent mice [Figure 3I]. IHC experiments showed that MC38-sh-circNCOA3-derived tumors presented a high amount of CD8^+^, but not CD4^+^, tumor-infiltrating lymphocytes (TILs) [Figure 4A and B, Supplementary Figure 1] and reduced infiltration of myeloid-derived suppressor cells (MDSCs) [Figure 4C and D, Supplementary Figure 1]. Furthermore, FC analysis revealed that circNCOA3 knockdown induced significantly more infiltration of CD8^+^ T cells and IFNγ^+^ cells in immune cells from tumors [Figure 4E and F, Supplementary Figure 2] but significantly reduced the amount of MDSCs and granulocytic (G)-MDSCs [Figure 4G and H, Supplementary Figure 2].

### CircNCOA3 functions as a molecular sponge for miR-203a-3p.1

**Figure 5:**
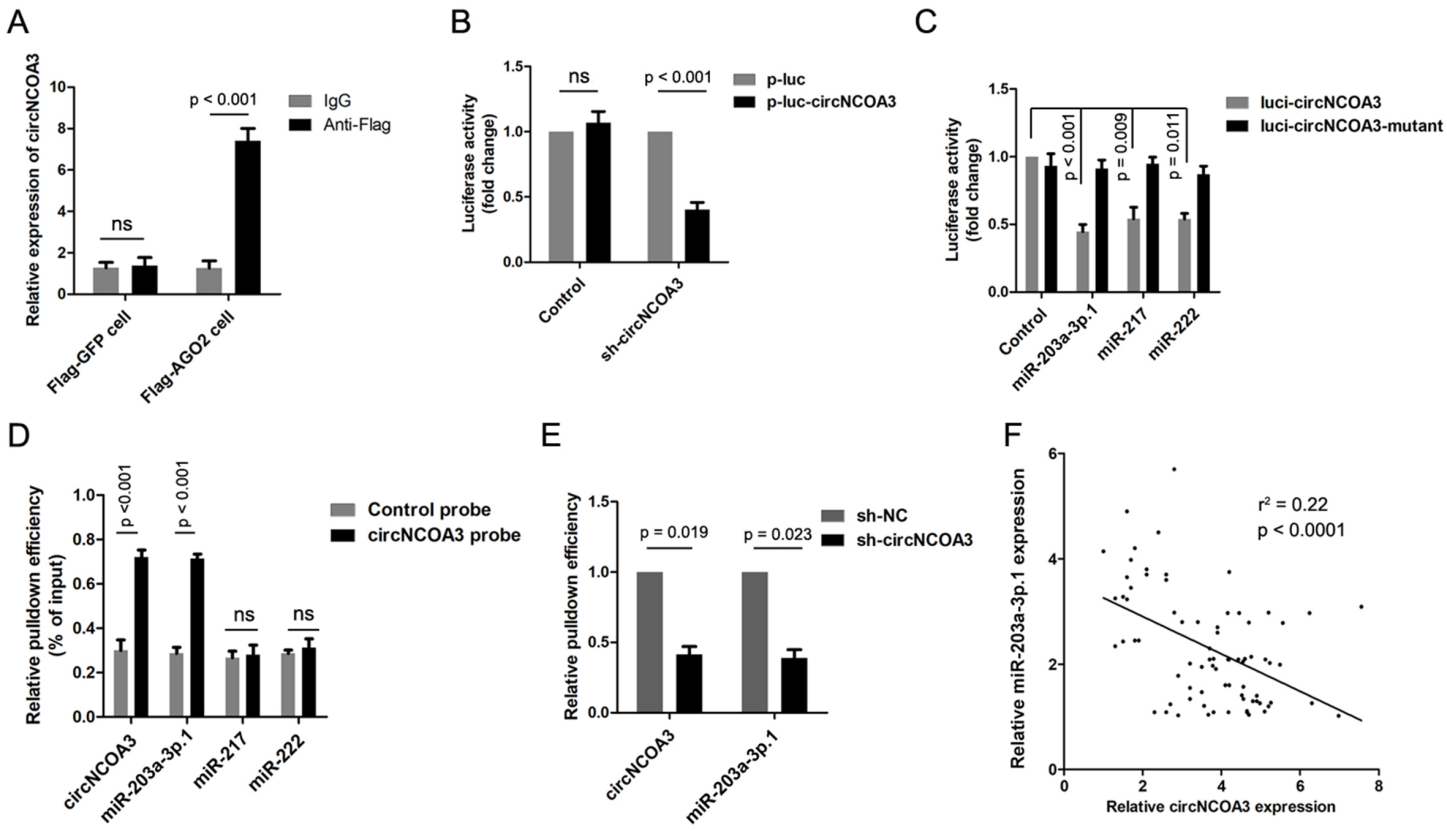
CircNCOA3 functions as a molecular sponge for miR-203a-3p.1. (A) RIP assays in CRC cells stably expressing Flag-AGO2 or Flag-GFP; (B) Luciferase activity of the circNCOA3 reporter in HCT116 cells transfected with circNCOA3 shRNA; (C) Luciferase activity of circNCOA3 reporter in CRC cells transfected with various miRNAs (miR-203a-3p.1, miR-217, miR-222); (D) RNA pulldown using a biotin-labeled circNCOA3 probe in CRC cells; (E) RNA pulldown using a biotin-labeled circNCOA3 probe in CRC cells transfected with sh-circNCOA3; (F) Correlation between the expression of circNCOA3 and miR-203a-3p.1 in 78 CRC tissues. RIP: RNA immunoprecipitation; CRC: colorectal cancer.

It is known that circRNAs generally function as competing endogenous RNAs (ceRNAs) for microRNAs to post-transcriptionally regulate the levels and dynamics of protein-coding transcripts. Because we found circNCOA3 primarily located in the cytoplasm, we hypothesized that this circRNA might exert its function through sponging certain miRNAs. RNA immunoprecipitation analysis showed endogenous circNCOA3 significantly enriched by Flag-AGO2 compared to Flag-GFP, indicating that circNCOA3 was incorporated in the RNA-induced silencing complex (RISC) [Figure 5A]. Using online bioinformatic tools, we identified several miRNAs that might be targeted by circNCOA3. To select the miRNAs that can be potentially regulated by circNCOA3, a circNCOA3 fragment was cloned and inserted into a luciferase reporter gene. circNCOA3 shRNA transfection significantly reduced the luciferase activity of the circNCOA3 reporter [Figure 5B]. The circNCOA3 reporter gene was co-transfected with the miRNA mimics into CRC cells. The result showed three miRNAs (miR-203a-3p.1, miR-217, and miR-222) significantly decreased the luciferase activity of the circNCOA3 reporter gene [Figure 5C]. To identify the potential miRNAs binding with circNCOA3, we performed RIP analysis using a biotin-labeled circNCOA3 probe, which showed that miR-203a-3p.1 was significantly enriched by the circNCOA3 probe, and the enrichment of miR-203a-3p.1 was reduced by knockdown of circNCOA3 in CRC cells [Figure 5D and E]. In addition, we found an inverse correlation between circNCOA3 and miR-203a-3p.1 expression in CRC tissues [Figure 5F].

### CXCL1 is the downstream target of miR-203a-3p.1

**Figure 6:**
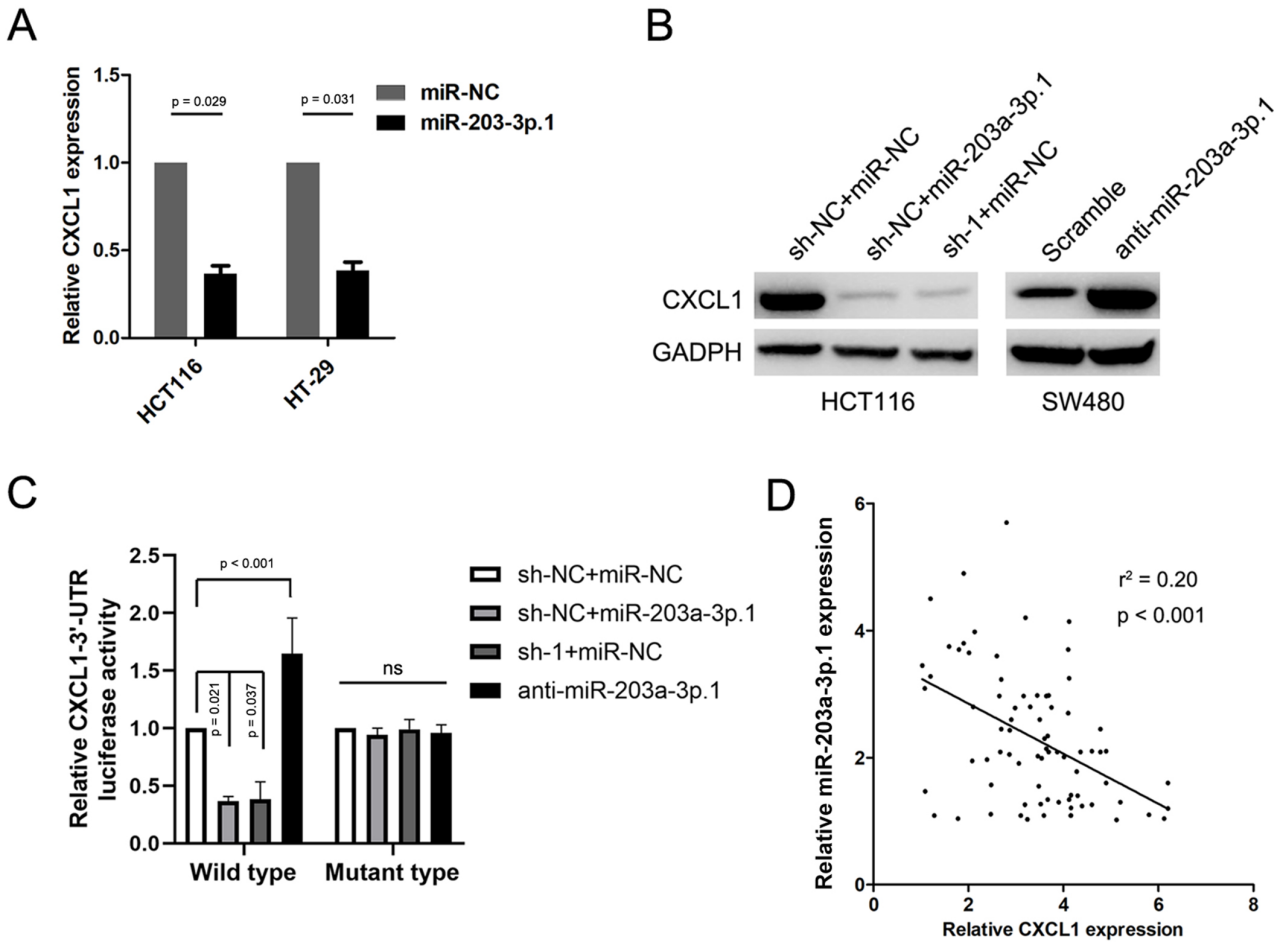
CXCL1 is the downstream target of miR-203a-3p.1. (A) RT-qPCR analysis of CXCL1 mRNA levels in CRC cells after miR-203a-3p.1 overexpression; (B) CXCL1 protein levels in CRC cells as detected by WB analysis; (C) Luciferase activity assay in CRC cells transfected with various vectors; (D) Correlation between the expression of miR-203a-3p.1 and CXCL1 in CRC samples. RT-qPCR: Real-time polymerase chain reaction; CRC: colorectal cancer; WB: Western blot.

The online bioinformatic tools TargetScan and miRanda were applied to identify the downstream targets of miR-203a-3p.1. CXCL1was selected as a key potential target of miR-203a-3p.1. We sought to validate the regulation of CXCL1 by miR-203a-3p.1. As expected, ectopic expression of miR-203a-3p.1 significantly reduced the mRNA levels of CXCL1 in CRC cells [Figure 6A]. Ectopic expression of miR-203a-3p.1 decreased CXCL1 protein levels, whereas inhibition of miR-203a-3p.1 had the opposite effect [Figure 6B]. Luciferase activity experiments showed that ectopic expression of miR-203a-3p.1 decreased the luciferase activity of the wild-type CXCL1 3’-UTR [Figure 6C]. Moreover, we also observed that the expression levels of miR-203a-3p.1 correlated negatively with those of CXCL1 in CRC samples [Figure 6D].

### Inverse correlation of CXCL1 and PD-1 antibody therapy response of CRC

**Figure 7:**
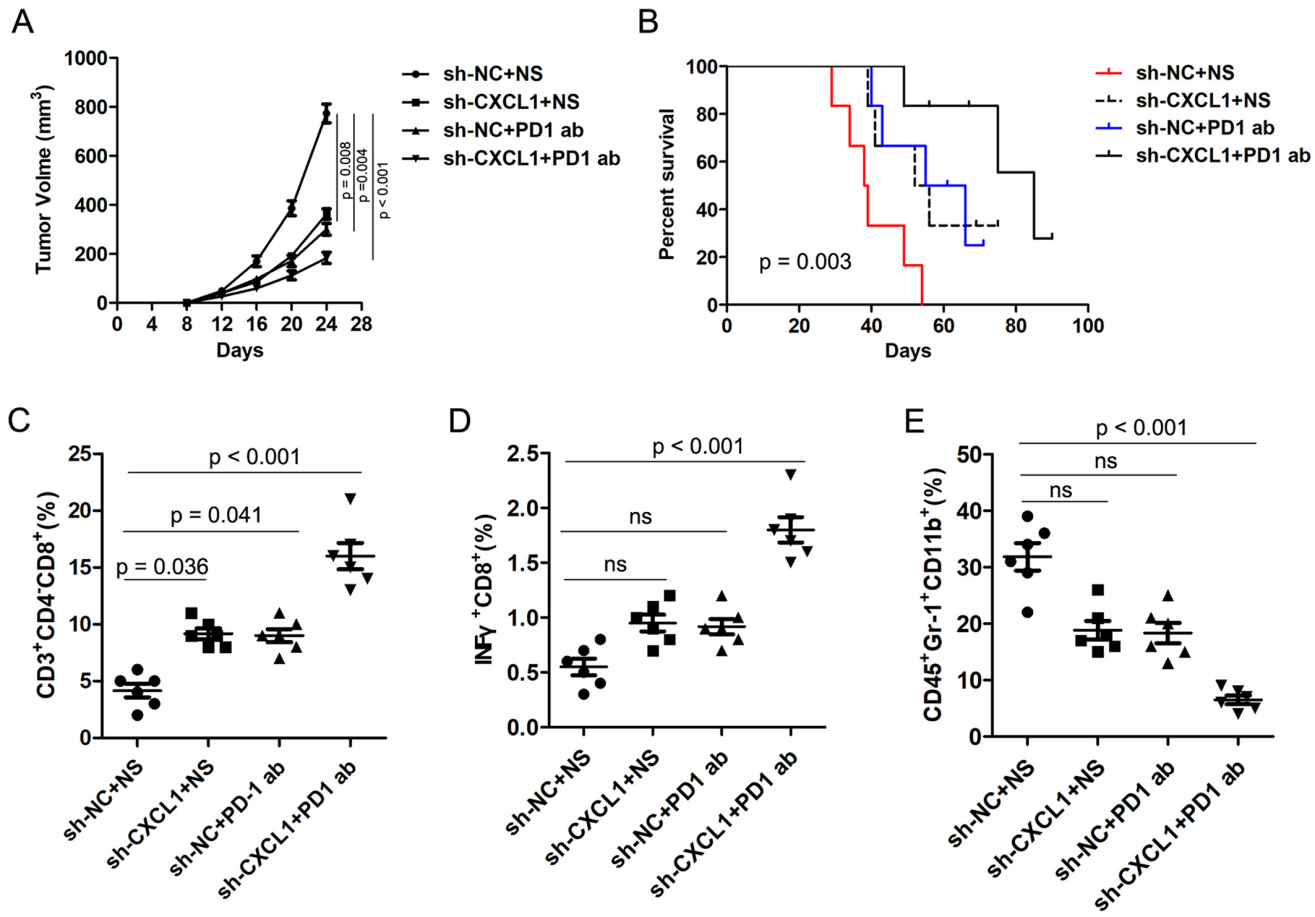
CXCL1 is negatively correlated with PD-1 antibody therapy responsiveness of CRC. (A) The volume of subcutaneous xenograft tumors was measured at indicated times in C57BL/6 mice; (B) The OS was analyzed by the Kaplan-Meier method in different groups; (C-E) The proportions of CD8^+^ T cells, INFγ^+^ cells, and MDSCs in different groups were detected by FC. PD-1: Programmed cell death 1; CRC: colorectal cancer; OS: overall survival; MDSCs: myeloid-derived suppressor cells; FC: flow cytometry.

Previous studies have reported that the CXCL1/CXCR2 axis is involved in tumor immune evasion. To investigate the role of CXCL1 on anti-PD-1 efficacy in CRC, MC38-sh-NC and MC38-sh-CXCL1 cells were injected subcutaneously into C57BL/6 mice. The mice were treated with PD-1 antibody and 0.9% normal saline (NS) was used as control. Interestingly, CXCL1 knockdown significantly inhibited tumor growth compared with the control group, and combined treatment with PD-1 antibody further inhibited tumor growth [Figure 7A]. Moreover, Kaplan-Meier analysis revealed that the knockdown of CXCL1 significantly prolonged the survival time of mice, and the combination with PD-1 antibody treatment synergistically prolonged the survival time of mice [Figure 7B]. FC analysis of the tumor tissues revealed that knockdown of CXCL1 combined with PD-1 antibody treatment markedly augmented the amount of CD8^+^ T cells and IFNγ^+^ cells and reduced the abundance of MDSCs [Figure 7C-E]. These results indicate that CXCL1 is inversely correlated with PD-1 antibody responsiveness of CRC.

## DISCUSSION

A growing body of literature has shown that circRNAs play key roles in the development of various cancers^[27,28]^. In addition, many circRNAs display specific patterns of expression in some cells and tissues^[29]^, implying that they might play important roles in a variety of biological processes. Deregulation of circRNAs has been associated with multiple pathological processes such as neurological disorders, cardiac hypertrophy, and tumorigenesis^[28,30]^. Previous studies found several circRNAs associated with CRC progression and immune evasion. For instance, Ding *et al.* found that a tumor-suppressive molecular axis activates the type I IFN pathway, inducing antitumor immunity to suppress CRC^[31]^. However, whether circRNAs are involved in anti-PD-1 resistance of CRC remains elusive. In the present study, by performing high throughput microarray analysis, we identified a series of circRNAs that are deregulated in CRC tissues. CircNCOA3 was significantly overexpressed in CRC patients resistant to anti-PD-1-based treatment. Moreover, circNCOA3 levels significantly correlated with the PFS and OS in CRC patients.

We then investigated the biological roles of circNCOA3 in CRC and found that knockdown of circNCOA3 significantly suppressed CRC cell proliferative and invasive capabilities. In mouse models, knockdown of circNCOA3 significantly suppressed the growth of tumors implanted in immunocompetent mice, but not in immunodeficient mice, implying circNCOA3 is associated with immune evasion. Indeed, we further found that the knockdown of circNCOA3 decreased the number of MDSCs while increasing the amount of CD8^+^ T cells. Previous reports have shown that aggressive tumor characteristics were correlated with a suppressive immune environment and negatively associated with PD-1 antibody therapy responsiveness^[32,33]^.

Tumor immune evasion is a key factor in tumor progression and resistance to treatment. Previous studies indicated that cancer cells are able to suppress the cytotoxic functions of CD8^+^ T cells by recruiting Tregs, MDSCs, and CD4^+^ T cells^[34,35]^. Moreover, PD-L1, an immune checkpoint, can negatively regulate T cell function through binding to its receptors PD-1 or B7-1, resulting in immune evasion in tumors^[35]^. Immunotherapy has recently emerged as an innovation in cancer therapy^[7]^. Anti-PD-1 therapy has greatly improved the therapeutic efficacy in CRC with MSI-H. However, most of the CRC patients are MSS, and these patients are not sensitive to anti-PD-1 therapy. Previous studies have reported additional biomarkers that could be potentially useful predictors for anti-PD-1 efficacy in CRC patients, such as tumor mutation burden, PD-L1 expression, and Pold/Pole mutation^[36-38]^. However, more studies are needed to explore new biomarkers for the prediction of PD-1 antibody treatment responsiveness of CRC. In this study, we report that circNCOA3 is negatively correlated with PD-1 antibody responsiveness of CRC and the expression of circNCOA3 is not associated with PD-L1 expression. Previously, we found that circDLG1 contributes to tumor escape from immune surveillance during tumor development and is associated with PD-1 antibody therapy effectiveness in gastric cancer^[25]^.

Mounting evidence shows that circRNAs function in multiple ways, such as modulating gene expression, interacting with RNA-binding proteins, and sponging RNAs^[39-41]^. For instance, our previous study showed that circTNIK promoted gastric cancer progression through sponging of miR-138-5p to influence the level of ZEB2^[22]^. Zhou *et al.* showed that circ-FIRRE can interact with HNRNPC to promote esophageal squamous cell carcinoma development through stabilizing GLI2 mRNA^[42]^. Here, we showed that circNCOA3 is mostly localized in the cytoplasm of CRC cells, suggesting that circNCOA3 might function as a miRNA sponge. Moreover, a RNA pulldown assay showed that circNCOA3 was able to co-sediment with miR-203a-3p.1. A luciferase activity assay demonstrated the direct interaction between circNCOA3 and miR-203a-3p.1. In addition, a RT-qPCR analysis showed an inverse correlation between the expression of circNCOA3 and miR-203a-3p.1.

**Figure 8:**
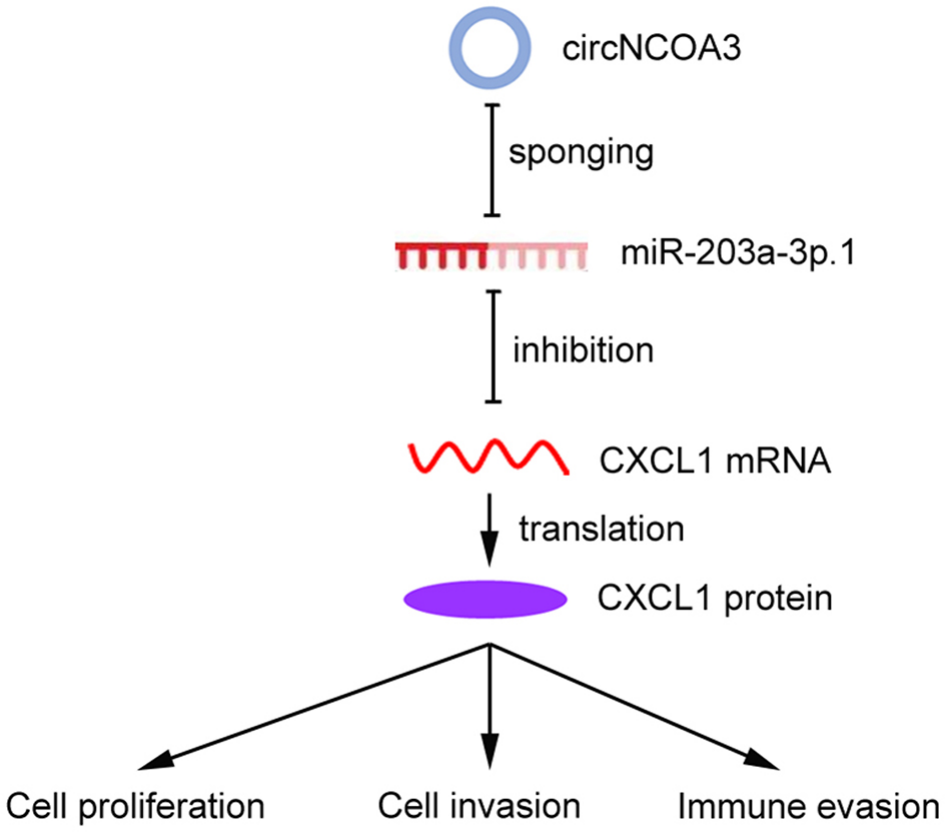
Working model of the mechanism of circNCOA3 on CRC progression and PD-1 antibody treatment resistance. CircNCOA3 acts as a ceRNA by competitively sponging miR-203a-3p.1 to regulate the levels of CXCL1 and modulate the proliferation, invasion, and immune evasion of CRC cells. CRC: Colorectal cancer; PD-1: programmed cell death 1; ceRNA: competing endogenous RNA.

We then explored the mechanism of circNCOA3 in mediating CRC immune evasion and identified CXCL1 as the downstream target of miR-203a-3p.1. miR-203a-3p.1 overexpression significantly reduced the CXCL1expression levels, whereas miR-203a-3p.1 inhibition had the opposite effect. The luciferase activity experiments indicated that overexpression of miR-203a-3p.1 markedly reduced the luciferase activity of wild-type CXCL1-3’-UTR. Moreover, we observed an inverse correlation between miR-203a-3p.1 and CXCL1 expression levels. Furthermore, we found that CXCL1 was involved in the regulation of the tumor immune environment and PD-1 antibody responsiveness of CRC. Previously, the CXCL1/CXCR2 axis was found to play a key role in promoting MDSCs chemotaxis in the tumor environment^[43]^. MDSCs could exert an immunosuppressive effect by inhibiting the proliferation and activation of key effector cells^[44]^. MDSCs are able to inhibit the function of antitumor T cells by multiple mechanisms, including induction of oxidative stress via iNOS, depletion of intratumoral arginine via Arginase-1, and secretion of TGF-β^[44]^. In line with our results, previous studies showed that the CXCL1/CXCR2 axis is involved in the antitumor immunity of CRC^[45]^. Taken together, these results indicated that circNCOA3 is involved in anti-PD-1 therapy resistance by regulating the miR-203a-3p.1/CXCL1 axis. Targeting this axis may potentially strengthen the effectiveness of PD-1 antibody treatment of CRC [Figure 8].

## DECLARATIONS

### Availability of data and materials

All data generated or analyzed in this study are included in this manuscript.

### Copyright

© The Author(s) 2024.
