# Supplementary material for: Circular RNA circNCOA3 promotes tumor progression and anti-PD-1 resistance in colorectal cancer
Source: Cancer Drug Resist. 2024 Mar 13;7:9. doi: 10.20517/cdr.2023.151 (PMC10951830; doi:10.20517/cdr.2023.151)
Supplement: Supplementary file 1 [file cdr-7-9-SupplementaryMethods.pdf]

Chen *et al*. *Cancer Drug Resist* 2024;7:9
DOI: 10.20517/cdr.2023.151

Cancer Drug Resistance

Supplementary Methods

Circular RNA circNCOA3 promotes tumor progression and anti-PD-1 resistance in colorectal cancer

Dong-Liang Chen, Nuo Chen, Hui Sheng, Dong-Sheng Zhang

State Key Laboratory of Oncology in South China, Guangdong Provincial Clinical Research Center for Cancer, Sun Yat-sen University Cancer Center, Guangzhou 510060, Guangdong, China.

Correspondence to: Prof. Dong-Liang Chen, State Key Laboratory of Oncology in South China, Guangdong Provincial Clinical Research Center for Cancer, Sun Yat-sen University Cancer Center, No. 651 Dong Feng East Road, Guangzhou 510060, Guangdong, China. E-mail: chendl@sysucc.org.cn

RNA sequencing

Total RNA was extracted form tissues with TRIzol according to the provider's instructions. The RNA was purified and concentrated with an RNeasy MinElute Cleanup kit (Qiagen, Valencia, CA, USA). RNA concentration and quality was evaluated with a NanoRrop ND1000 spectrophotometer (NanoDrop, Wilmington, DE, USA). Then, the electrophoresis on a denaturing agarose gel was performed to detect the RNA integrity and DNA contamination. The RNA was treated with RNase R to remove linear RNAs and enrich pure circRNAs. The RNA-seq libraries were performed using pretreated RNAs with TruSeq Stranded Total RNA Library Preq Kit (Illumina, San Diego, CA, USA) following the manufacturer's instructions. Sequencing was performed by using an Illumina HiSeq 4000 Sequencing System for 150 cycles (KangChen Bio-tech Inc, Shanghai, China). HTSeq software was used to count the reads numbers mapped to circRNA. Reads Per Million mapped reads (RPM) was employed to calculate the expression of individual circRNA. Differentially expressed RNAs were evaluated by using the DEGseq algorithm. The differentially expressed circRNAs were defined as q-value < 0.05 with a fold change > 2 or < 0.5.

Quantitative real-time polymerase chain reaction (RT-qPCR)

Total RNA was isolated from human CRC tissues, serum, and cell lines by using the Trizol reagent (Sigma, St. Louis, MO, USA) according the manufacturer's instructions. Reverse transcription of mRNA and miRNA was performed using random primers and stem-loop primers, respectively. Real-time PCR was conducted by using a TaqMan Universal Master Mix II kit on a Bio-Rad CFX96 qPCR system, fold changes were determined by using the relative quantification $2^{-\triangle\triangle CT}$ method. The nuclear and cytoplasm fractions of cells were separated by using the PARIS Kit (Life Technologies) according to the manufacturer's instructions. RNA was extracted from both fractions. Then, real-time PCR was performed to determine the expression ratios of specific RNA molecules between the nuclear and cytoplasm fractions. GADPH and U6 served as the cytoplasm and nuclear markers, respectively.

Cell proliferation assays

To evaluate the cell proliferation, the cell counting kit-8 (CCK-8) and colony formation assays were used. For CCK-8 assay, cells (1×10^3) were cultured in a

96-well plate at 37 °C. The plates were incubated at 37 °C for 2 h after 10 μL CCK-8 solutions were added to each well. The spectrophotometric absorbance at 450 nm was measured for each sample. All the experiments were repeated 3 times in triplicate and the mean was calculated. For colony formation assay, cells were trypsinized and suspended in culture medium. The cells were seeded in 6-well plates and cultured in a humidified atmosphere containing 5% CO_2 at 37 °C for 2 weeks. Cell colonies were washed with PBS, fixed with methanol, and stained with 0.1% crystal violet (1 mg/mL). Colonies containing more than 50 cells were counted and the mean colony numbers were calculated.

Cell invasion assay

Transwell assay was used for evaluating the cell invasion ability. Briefly, cells were trypsinized and 1×10^5 cells in 100 μL of serum-free RPMI-1640 medium were plated into the upper chamber. RPMI-1640 medium (500 μL) supplemented with 20% FBS was added to the lower chamber. After culturing for 22 h, cells that had invaded the lower chamber were fixed with methanol and stained with 0.1% crystal violet. The number of invaded cells was observed by using inverted microscope (magnification × 200) and calculated by counting five random views.

RNA immunoprecipitation (RIP) and luciferase activity assays

The RIP assay was performed by using the Magna RIP RNA-bide Protein Immunoprecipitation kit (Millipore, USA) following the provider's instructions. Briefly, cell lysates were cultured with Dynabeads-coated IgG antibody (Millipore, USA) or AGO2 antibody (Cell Signaling Technology, USA) for 12 h at 4 °C. The purified RNA was subjected to qRT-PCR to detect the enriched circNCOA3 and miRNA. For the luciferase activity assay, potential binding sites were predicted using StarBase v3.0 and TargetScanHuman. Cells were cotransfected with pGL-luc-circNCOA3, pGL-luc-CXCL1 3'-UTR, and miR-203a-3p.1 mimics or miR-203a-3p.1 inhibitors for 48 h, and luciferase activity was detected using the dual-luciferase reporter assay system (Promega, USA) according to the manufacturer's instructions.

***In vivo* tumorigenesis assay**

MC38-sh-circNCOA3 and MC38-sh-NC cells (1×10^6 cells/mouse) were inoculated

subcutaneously into the flanks of two groups of BALB/c nude mice and C57BL/6 mice (ten for each cell group). Tumor size was measured every 4 days, and tumor volume was estimated. The tumors were excised after the mice were dead.
