# Supplementary material for: Circular RNA circNCOA3 promotes tumor progression and anti-PD-1 resistance in colorectal cancer
Source: Cancer Drug Resist. 2024 Mar 13;7:9. doi: 10.20517/cdr.2023.151 (PMC10951830; doi:10.20517/cdr.2023.151)
Supplement: Supplementary file 2 [file cdr-7-9-SupplementaryFiguresandTables.pdf]

Chen *et al. Cancer Drug Resist* 2024;7:9
DOI: 10.20517/cdr.2023.151

Cancer
Drug Resistance

Supplementary Figures and Tables

Circular RNA circNCOA3 promotes tumor progression and anti-PD-1 resistance in colorectal cancer

Dong-Liang Chen, Nuo Chen, Hui Sheng, Dong-Sheng Zhang

State Key Laboratory of Oncology in South China, Guangdong Provincial Clinical Research Center for Cancer, Sun Yat-sen University Cancer Center, Guangzhou 510060, Guangdong, China.

Correspondence to: Prof. Dong-Liang Chen, State Key Laboratory of Oncology in South China, Guangdong Provincial Clinical Research Center for Cancer, Sun Yat-sen University Cancer Center, No. 651 Dong Feng East Road, Guangzhou 510060, Guangdong, China. E-mail: chendl@sysucc.org.cn

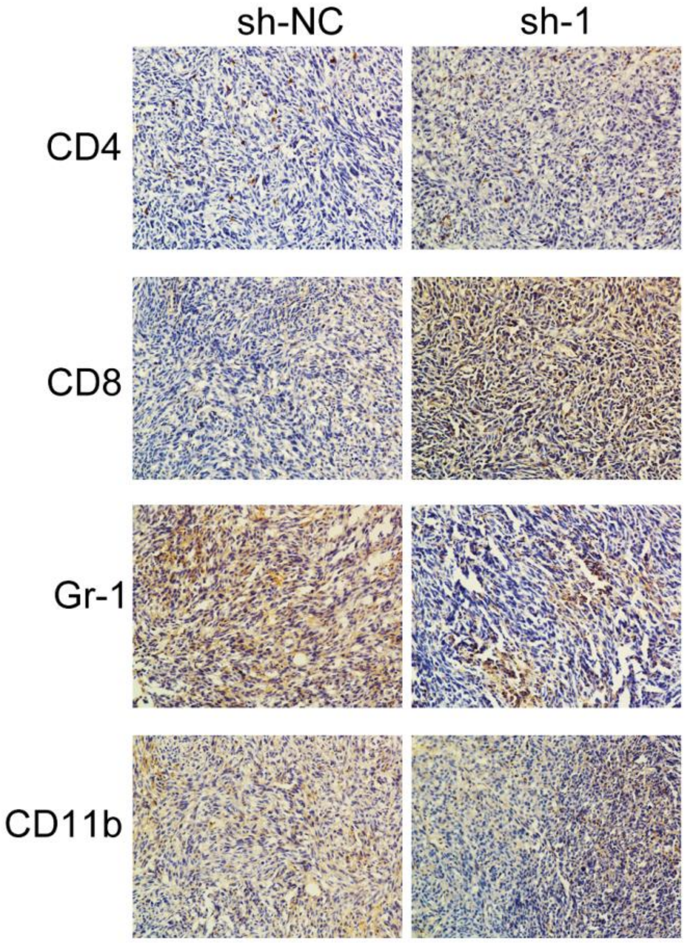

Supplementary Figure 1. The representative images of IHC for CD4, CD8, Gr-1, and CD11b in different groups.

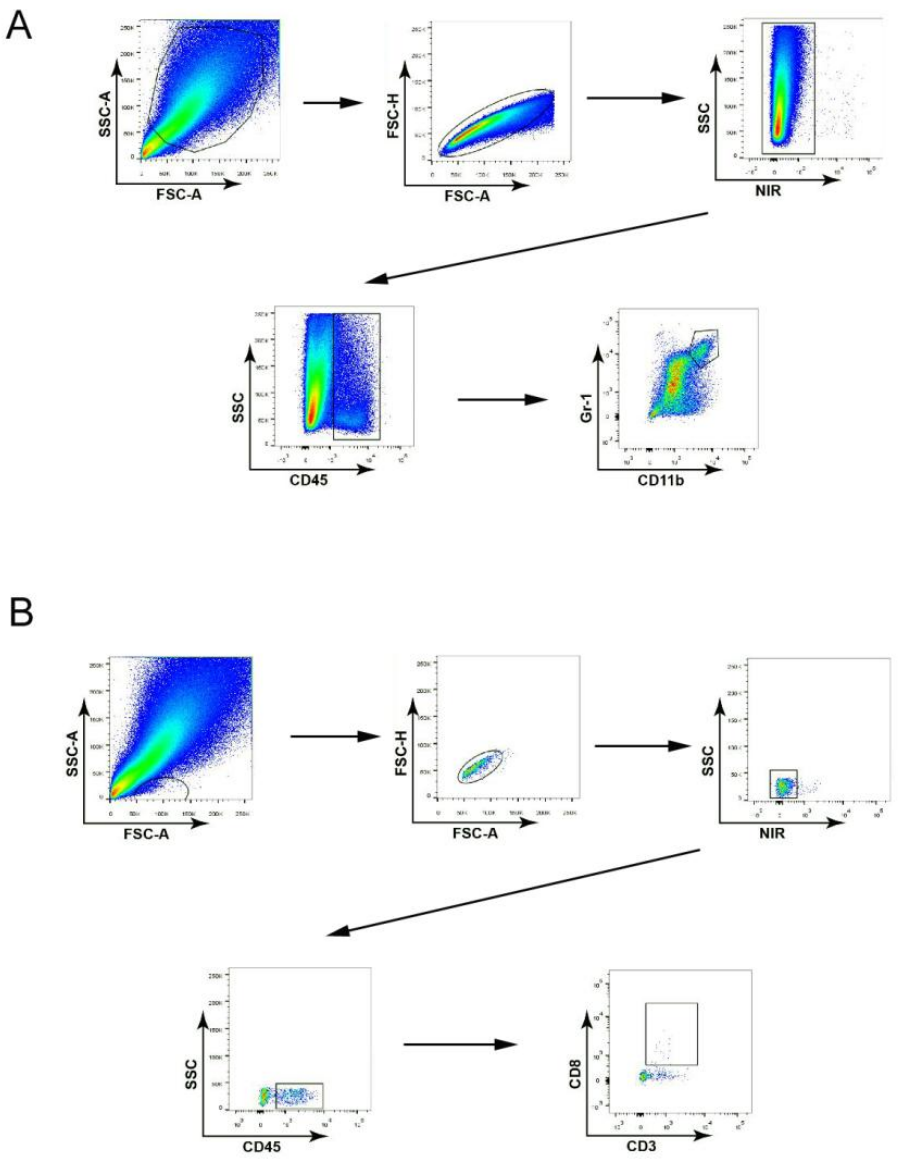

Supplementary Figure 2. The gating strategy of Flow cytometry for MDSC (A) and CD8 T cells (B).

Supplementary Table 1. Primers used in the paper were listed

Gene	Primer	Sequence(5’-3’)
**Primers for qRT-PCR**		
Circ_0060627	Forward	5’-CACGCCGCATTACTACAGGA-3’
	Reverse	5’-CACCACTGCAGGTAAGACTGAA-3’
GAPDH	Forward	5’-TGCACCACCAACTGCTTAGC-3’
	Reverse	5’-GGCATGGACTGTGGTCATGAG-3’
U6	Forward	5’-CTCGCTTCGGCAGCACA-3’
	Reverse	5’-AACGCTTCACGAATTTGCGT-3’

Supplementary Table 2. The information for antibodies used in flow cytometry

Antibodies	Company	Clone/catalog number
Anti-mouse F4/80	BioLegend	BM8
Anti-mouse CD45	BD Biosciences	30-F11
Anti-mouse IFNGγ	BD Biosciences	XMG1.2
Anti-mouse Gr-1	TONBO Biosciences	RB6-8C5
Anti-mouse Ly6G	TONBO Biosciences	1A8
Anti-mouse Ly6C	BD Biosciences	AL-21
Anti-human CD33	BD Biosciences	WM53
Anti-human CD11b	TONBO Biosciences	M1/70
Anti-human HLA-DR	BD Biosciences	G46-6

Supplementary Table 3. The correlation between clinicopathologic parameters and circNCOA3 expression in 55 CRC patients

**Characteristics**	***n***	**High expression**	**Low expression**	***P* value**
Age				0.508
< 60	24	11	13	
≥ 60	31	17	14	
Gender				0.343
Male	36	20	16	
Female	19	8	11	
Tumor size				0.010
< 5 cm	29	10	19	
≥ 5 cm	26	18	8	
Tumor cell Differentiation				0.091
Well	7	2	5	
Moderate	26	11	15	
Poor	22	15	7	
Tumor location				0.079
Left colon/rectum	27	17	10	
Right colon	28	11	17	
Liver metastasis				0.004
Present	30	20	10	
Absent	25	7	18	

Chi-square test.
